# Pericardial Metaplastic Ossification With Widespread Bone Formation: A Case Report

**DOI:** 10.1155/crip/4462684

**Published:** 2026-03-03

**Authors:** B. Gharia, A. Graham, McEwen C. C, R. P. Whitlock

**Affiliations:** ^1^ Department of Pathology and Molecular Medicine, Hamilton General Hospital, McMaster University, Hamilton, Ontario, Canada, mcmaster.ca; ^2^ Department of Cardiac Surgery, Hamilton General Hospital, Hamilton, Ontario, Canada, hamiltonhospital.org

## Abstract

Pericardial disease can present clinically as acute pericarditis, pericardial effusion, cardiac tamponade, and constrictive pericarditis (CP). Pericardial calcification is present in less than 25% of all cases of CP, and patients with this finding are at risk for developing additional cardiac complications. A case of extensive pericardial ossification in a 65‐year‐old male presenting with pericardial effusion is reported. The patient had several episodes of pericardial effusion and was diagnosed with CP and subsequently managed on medical therapy. Due to worsening symptoms, additional investigations were completed, including echocardiogram, which revealed diastolic dysfunction with constrictive physiology. He underwent bilateral anterior pericardiectomy, and during the surgery, thickened, calcified, and adherent pericardium was identified, along with pockets of effusion. Cytology of pericardial fluid was negative for malignant cells. Histologic sections of the pericardium demonstrated extensive pericardial ossification with widespread bone formation in pericardial/epicardial adipose tissue. Idiopathic pericardial ossification, as identified in our case, is a rare phenomenon only described several times in the literature and may cause CP. Early intervention with pericardiectomy is a predictor of good outcome in CP, and thus, it is important to include rare entities such as metaplastic ossification in the differential for pericardial disease, to facilitate prompt diagnosis and surgical intervention.

## 1. Introduction

The pericardium is a rigid, avascular, fibrous sac approximately 1–2 mm thick and is composed of an outer fibrous layer and an inner serous layer (which further subdivides into a visceral layer, or epicardium, and a parietal layer) [1]. A potential space that contains approximately 15–35 mL of lubrication fluid separates the visceral and parietal layers [1]. The primary function of the pericardium is anchoring and protection of the heart, lubrication, preventing distention of cardiac chambers, and optimizing diastolic filling [2].

Pericardial disease often begins with a constellation of symptoms such as chest pain, dyspnea, and fatigue [3]. Pericardial diseases include pericardial effusion, cardiac tamponade, and inflammatory diseases, which constitute a spectrum ranging from acute pericarditis to chronic constrictive pericarditis (CP) [4, 5]. CP occurs as a result of scarring and consequent decrease in elasticity of the pericardial sac and is characterized by adhesion and inflammation of the pericardium that may result in heart failure [6]. CP can occur after any pericardial disease process but often follows acute pericarditis or cardiac surgery [6]. Calcification and thickening of the pericardium are pathologies unique to chronic CP [6].

Pericardial calcification is present in less than 25% of all cases of CP and is usually of varied etiology, including chronic inflammation and genetic predisposition [7]. Pericardial ossification, a rare and intriguing phenomenon, involves abnormal deposition of calcium within the pericardium, leading to the formation of bone‐like structures that may compromise the heart′s ability to contract and relax [3, 8]. This condition is distinct from common pericardial diseases and often presents a diagnostic challenge due to its infrequency and varied clinical manifestations [8].

The nonspecific nature of symptoms in pericardial disease necessitates a comprehensive diagnostic approach, including imaging modalities such as echocardiography, computed tomography (CT) scans, and magnetic resonance imaging (MRI) [3]. Despite advances in diagnostic techniques, distinguishing pericardial ossification from other pericardial disorders remains challenging, thus emphasizing the need for a multidisciplinary approach involving cardiologists, radiologists, and pathologists [3, 9].

Treatment strategies for CP due to pericardial ossification are not well established, given the scarcity of reported cases [8]. Symptomatic management often involves addressing the consequences of pericardial ossification, such as heart failure or conduction abnormalities [3]. Surgical intervention is considered in severe cases where the calcified deposits significantly impede cardiac function [3]. Clarifying underlying mechanisms of pericardial calcification is important in developing targeted therapeutic approaches and preventive measures for associated conditions such as atrial fibrillation [7].

This case report describes the clinical presentation, investigations, pathology, and management strategies associated with pericardial ossification and is aimed at adding to the existing body of knowledge on pericardial ossification, including clinical nuances and potential therapeutic avenues.

## 2. Case Presentation

A 65‐year‐old man with a history of hypercholesterolemia, Type 2 diabetes mellitus, COPD, smoking, and opioid dependence presented to the Emergency Department in Hamilton, Ontario, Canada, in February 2021. He was experiencing progressive exertional dyspnea, chest discomfort, and worsening fatigue and was admitted to the cardiology intensive care unit. Cardiovascular exam revealed an internal jugular cordis on the right side, ECG showed normal sinus rhythm with subtle ST depression in V3–V5, and CT chest/abdomen/pelvis showed no evidence of malignancy. A large pericardial effusion was identified, and the patient subsequently underwent pericardiocentesis with drain placement. Cytologic assessment as well as bacterial, fungal, and mycobacterial cultures of the pericardial fluid were negative, and the effusion was deemed idiopathic. Flow cytometry completed at that time was also noncontributory. Follow‐up echocardiography in March 2021 demonstrated a smaller residual effusion with features of CP, and right heart catheterization completed in October 2021 demonstrated constrictive physiology with compensated filling pressures. The patient was stable on medical therapy with colchicine (commenced in February 2021) but continued to report exertional dyspnea, with worsening of symptom burden over the next 6 months. He was referred for surgical management in May 2022.

The patient underwent a bilateral anterior pericardiectomy, during which an extensively calcified pericardium was observed and meticulously excised to restore cardiac mobility; the posterior thickened pericardium was left intact. Excised pericardial tissue, along with pericardial fluid, was sent to pathology for analysis. The postoperative course was complicated by COPD exacerbation, bilateral pleural effusion, and uncontrolled atrial fibrillation; the patient subsequently received a complete amiodarone load. At the time of discharge, he was started on warfarin and followed by the thrombosis service.

## 3. Results

**Figure 1:**
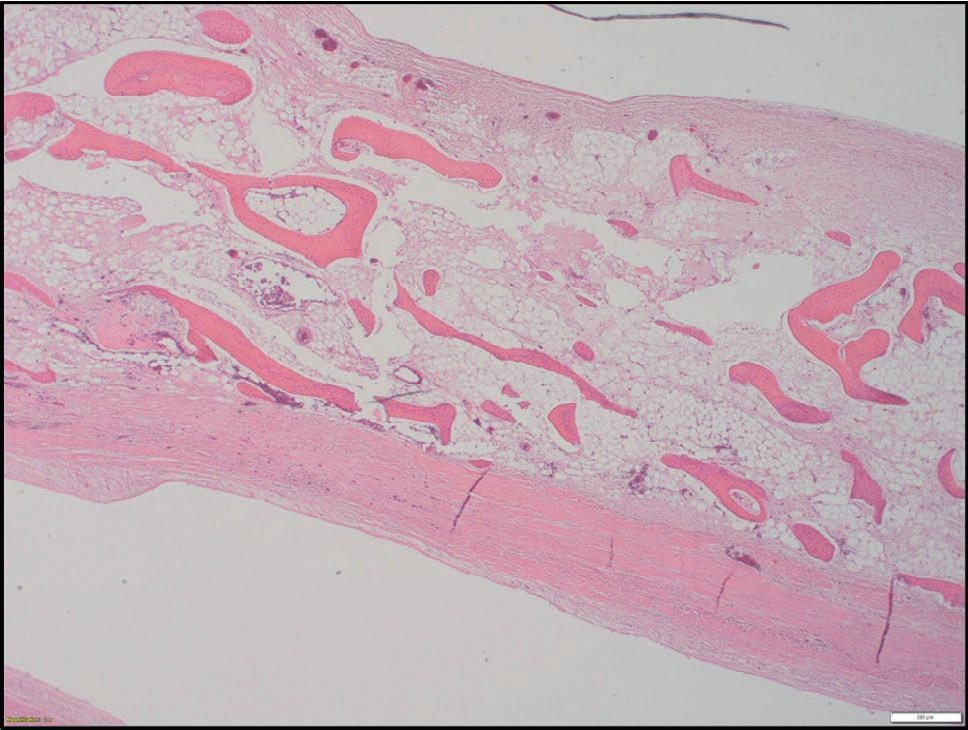
Extensive pericardial metaplastic ossification showing numerous bony trabeculae (H&E, low‐power view).

**Figure 2:**
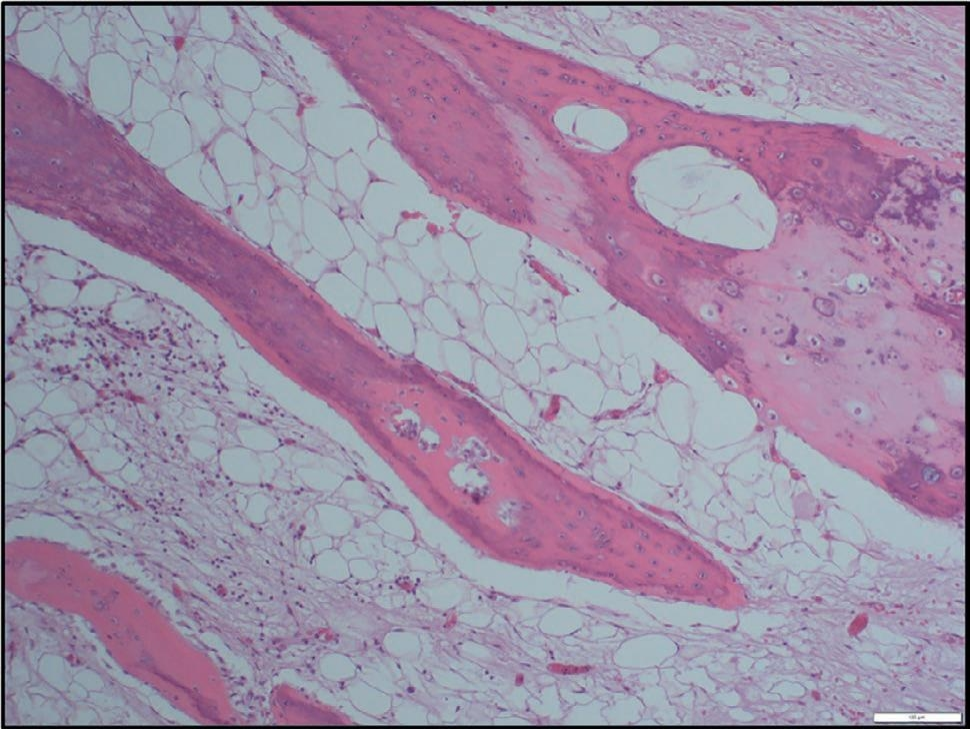
Osteoblastic rimming in bone formation, pericardium (H&E, medium‐power view).

Pericardial tissue received by pathology measured 8.0 × 6.5 cm, with thickened areas measuring up to 0.3 cm. Tissue appeared congested and moderately calcified; tissue was decalcified prior to processing, and all pericardial tissue was submitted for microscopic examination. On histologic sections, about 85%–90% of tissue showed widespread, extensive pericardial metaplastic ossification wherein numerous bony trabeculae were observed throughout pericardial adipose tissue (Figure 1). Bony trabeculae consisted primarily of woven bone with osteoblastic rimming and scattered foci of mature lamellar bone (Figure 2). Trilineage hematopoiesis was observed within trabecular marrow spaces in numerous areas, primarily erythroid and myeloid precursors, along with abundant mature adipose tissue (Figure 3a,b). Of note, ossification was restricted to the pericardial adipose tissue and was not seen in fibrous pericardium. Sections without ossification demonstrated moderate to severe thickening of fibrous pericardium with scattered nonspecific inflammation. No granulomatous inflammation or cholesterol deposits were observed. Special stains for organisms, including gram stain for intracytoplasmic bacteria, GMS stain for fungi, and Ziehl–Neelsen stain for acid‐fast bacteria, were negative.

Figure 3(a) Pericardial metaplastic ossification, showing mature adipose tissue and hematopoietic elements of all three cell lineages (H&E, medium‐power view). (b) Pericardial metaplastic ossification, showing mature adipose tissue and hematopoietic elements of all three cell lineages between bony trabeculae (H&E, high‐power view).

**Figure:**
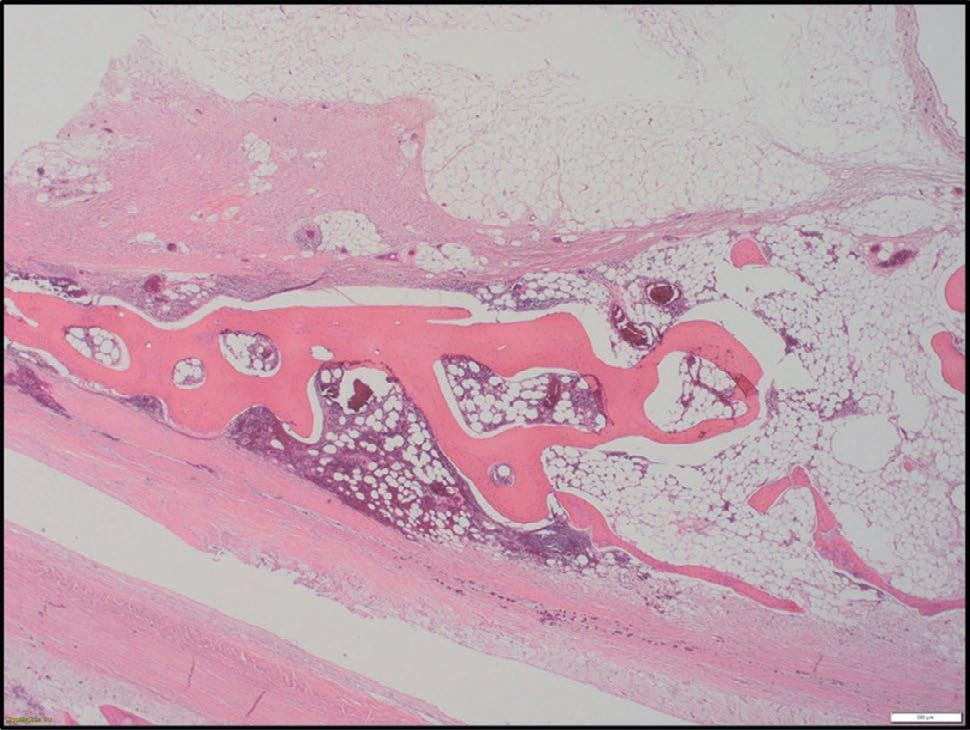
(a)

**Figure:**
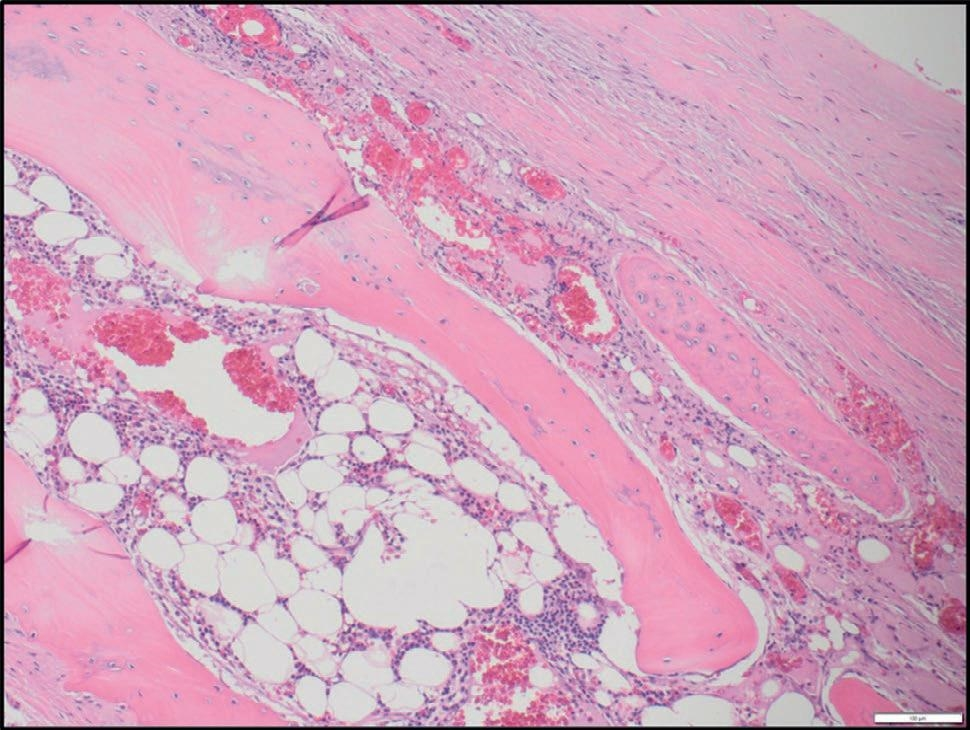
(b)

Cytology of pericardial fluid showed reactive mesothelial cells with a hyperplastic appearance and numerous histiocytic cells engulfing red blood cells and was negative for malignant cells.

## 4. Discussion

Normal pericardium lacks calcium deposits, and calcification may be a sign of underlying inflammation or a more sinister etiology [2]. Pericardial calcifications are usually deposited in regions of pericardial inflammation and fibrosis, which may be caused by tuberculous, fungal, viral, or pyogenic infections; trauma and hemopericardium; cardiac surgery; and collagen vascular diseases such as lupus, rheumatic heart disease, uremic pericarditis, and radiation [10]. Hypothyroidism may also cause calcific pericardial disease and is usually associated with cholesterol deposits, which were not observed in the current specimen [10]. Tuberculosis (TB) has historically been the leading cause of pericardial calcifications worldwide; however, in our case, TB and fungal infections were ruled out with special stains [11].

Idiopathic pericardial ossification may occur in apparently healthy individuals and, in rare cases, may cause chronic CP [12]. This phenomenon has only been observed several times in the literature, most recently in a 17‐year‐old male patient who presented with pericardial effusion and evidence of CP on imaging; histology demonstrated formation of woven and lamellar bone, primarily in the fibrous pericardium [12]. Literature search also revealed a related entity described in chimpanzees with myocardial fibrosis; chimps may develop ossa cordis, described as small bones within the cardiac skeleton (particularly in fibrous trigones), which are thought to aid in maintaining the heart′s shape during systole and ensure cardiac contraction efficiency [13, 14]. Recently, ossa cordis was reported for the first time in a human heart [15].

Some individuals may be genetically predisposed toward bone formation and show excessive osteophytosis; metaplastic ossification observed in this case could possibly be associated with a natural progression of exuberant calcification. This patient′s initial pericardial effusion and pericardiocentesis are not considered direct causes of pericardial ossification; however, inflammation related to the pericardial effusion in 2021 and evolution into CP can establish a sustained inflammatory and fibrotic environment that may predispose to calcification and, in rare cases, ossification [2]. Consequently, further correlation with laboratory investigations, including serum calcium and parathyroid hormone, was suggested for this patient.

Early intervention with pericardiectomy is a predictor of good early and late outcome in CP [7]. Prolonged constriction can result in myocardial atrophy, residual constriction, and persistent heart failure despite successful pericardiectomy [7].

This case report provides a detailed account of clinical presentation, investigations, management, and pathologic examination in a rare case of pericardial ossification. Lack of clear etiological factors in this case emphasizes the need for further research to unravel underlying mechanisms triggering abnormal calcification within the pericardium. The rarity of pericardial ossification underscores the importance of heightened clinical suspicion, especially in patients presenting with atypical cardiac symptoms. As more cases are encountered, collaborative efforts across medical disciplines will be essential to enhance our understanding of and refine diagnostic criteria of this rare condition and ultimately guide therapeutic strategies and improve long‐term outcomes for patients.

## Funding

No funding was received for this manuscript.

## Consent

The patient in our case report allowed personal data processing, and informed consent was obtained from this individual participant and included in the study.

## Conflicts of Interest

The authors declare no conflicts of interest.

## Data Availability

The data that support the findings of this study are available on request from the corresponding author. The data are not publicly available due to privacy or ethical restrictions.
